# Microbiota–immune crosstalk in the regulation of intestinal motility in constipation

**DOI:** 10.3389/fmicb.2026.1828926

**Published:** 2026-04-21

**Authors:** Zhengchao Pan, Tao Zhang, Zhetan Ren, Hongkun Zhang, Jing Wang, Yongxun Ma, Ru Man, Jirun Peng, Yongduo Yu

**Affiliations:** 1 Liaoning University of Traditional Chinese Medicine, Shenyang, China; 2 Second Affiliated Hospital, Liaoning University of Traditional Chinese Medicine, Shenyang, China; 3 Department of General Surgery, Beijing Shijitan Hospital, Capital Medical University, Beijing, China; 4 Ninth School of Clinical Medicine, Peking University, Beijing, China

**Keywords:** constipation, gut microbiota, gut microecology, immune regulation, intestinal motility

## Abstract

Constipation is a common functional gastrointestinal disorder with a complex pathogenesis. Traditional studies have primarily explained its development in terms of reduced intestinal motility or impaired defecatory coordination; however, these mechanisms alone cannot fully account for the multifactorial pathological processes underlying the condition. In recent years, increasing attention has been directed toward the roles of intestinal microbial dysbiosis and alterations in immune homeostasis in the development of constipation. The gut microbiota continuously interacts with the intestinal immune system through its structural components, metabolic products, and secreted molecules. These interactions modulate the mucosal immune microenvironment and participate in the regulation of intestinal motility by influencing the enteric nervous system, interstitial cells of Cajal, and smooth muscle function. Conversely, the immune system can reshape the composition and spatial distribution of the gut microbiota through mechanisms such as the mucosal barrier, immunoglobulin A, and antimicrobial peptides, thereby forming a bidirectional regulatory network. Accumulating evidence suggests that during the onset and progression of constipation, microbial dysbiosis, shifts in immune homeostasis, and abnormalities in intestinal motility may evolve through a progressively amplifying dynamic process, ultimately establishing a self-sustaining chronic cycle. In addition, microbiota-targeted interventions—including probiotics, prebiotics, and fecal microbiota transplantation—have demonstrated potential benefits in improving stool frequency and stool consistency in several clinical studies. However, the immunological mechanisms underlying these effects remain relatively underexplored. This review systematically summarizes the molecular mechanisms by which gut microbiota–immune interactions regulate intestinal motility. By integrating current evidence on disease progression and clinical studies, we propose a conceptual model of the “microbiota–immune–motility regulatory axis,” aiming to provide a new perspective for understanding the pathogenesis of constipation and for optimizing microbiota-based therapeutic strategies.

## 1 Introduction

Constipation is a common functional gastrointestinal disorder characterized by reduced bowel movement frequency, difficulty in defecation, and a sensation of incomplete evacuation. These symptoms can substantially impair patients’ quality of life and increase long-term healthcare burdens (Zhang J. et al., 2025; Dimidi, 2025). Traditionally, the pathogenesis of constipation has been explained in terms of reduced intestinal motility, delayed colorectal transit, or impaired defecatory coordination (Li J. et al., 2025). However, these mechanisms alone cannot fully account for the heterogeneity of constipation or its complex pathological processes. Recent studies suggest that the development and progression of constipation may also be associated with alterations in the intestinal microecological environment and disruptions in immune homeostasis (Xu et al., 2024; Lee S. et al., 2025).

The gut microbiota is a key determinant of intestinal homeostasis and plays an important role in regulating the mucosal barrier, immune responses, and the function of the enteric nervous system (Aburto and Cryan, 2024; Di Vincenzo et al., 2024). Multiple studies have reported alterations in both the composition and metabolic functions of the gut microbiota in individuals with constipation, including reduced capacity for short-chain fatty acid–related metabolism, changes in microbial diversity, and abnormal proportions of specific functional bacterial taxa (He et al., 2025). At the same time, the role of the intestinal immune system in constipation has gained increasing attention. Intestinal mucosal immune cells can recognize microbe-associated signals and release cytokines and inflammatory mediators, thereby contributing to the maintenance of immune tolerance and the regulation of inflammatory balance (Chi et al., 2025). Alterations in microbial composition and microbial metabolites may shift immune homeostasis, which in turn can influence the enteric nervous system, interstitial cells, and smooth muscle function, ultimately affecting intestinal motility (Yuan et al., 2024; de Vos et al., 2022).

**Figure 1:**
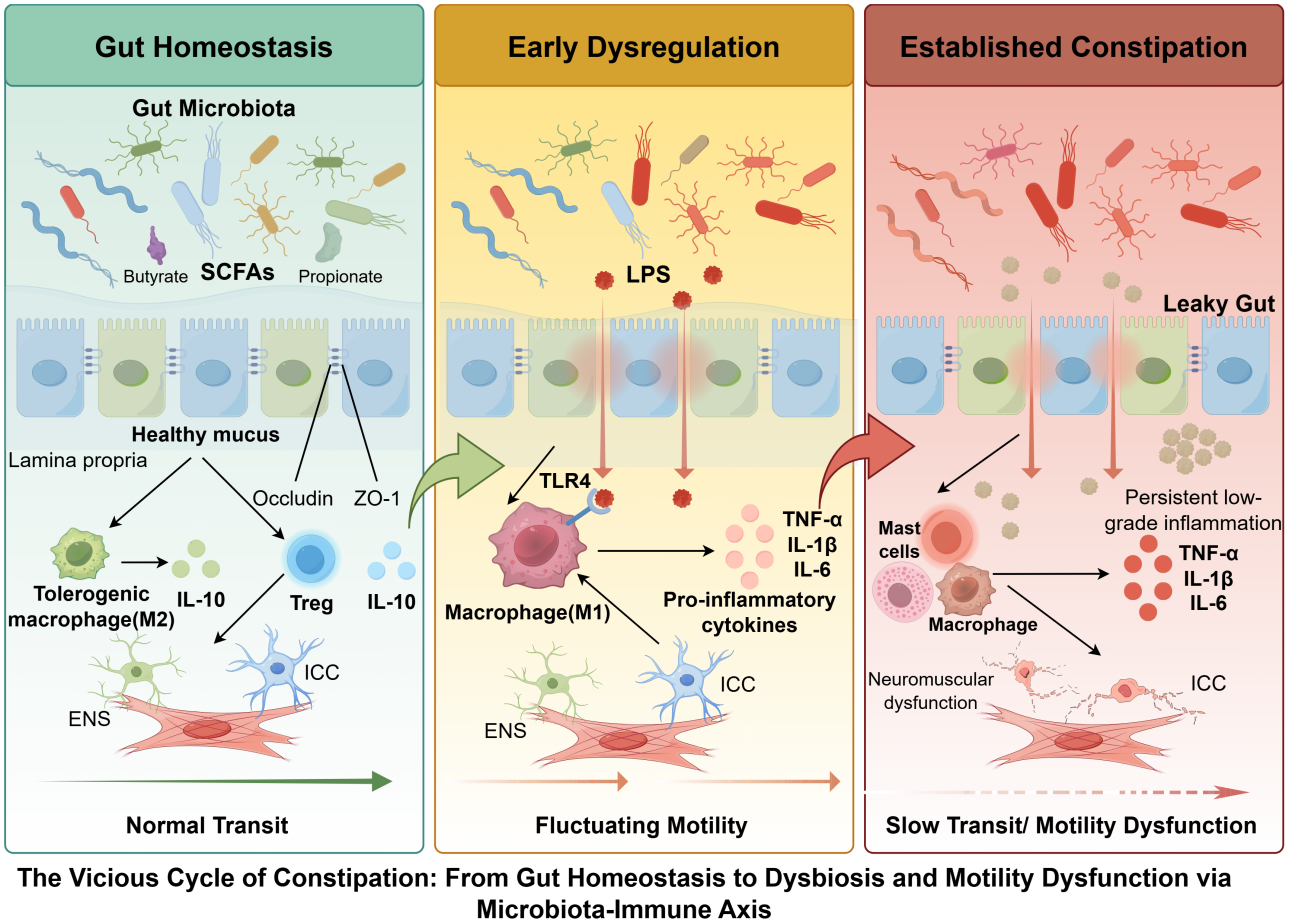
Conceptual framework of microbiota–immune–motility interactions in constipation. In gut homeostasis, short-chain fatty acids produced by the gut microbiota support epithelial barrier integrity and promote tolerogenic immune responses, thereby maintaining coordinated enteric nervous system (ENS), interstitial cells of Cajal (ICC), and smooth muscle function, resulting in normal intestinal transit. During early dysregulation, increased microbial-derived signals activate immune pathways, leading to macrophage polarization and elevated pro-inflammatory cytokines. These changes disrupt epithelial barrier integrity and interfere with neuromuscular regulation, resulting in fluctuating motility. In established constipation, persistent dysbiosis, barrier dysfunction, and chronic low-grade inflammation further impair ENS function, disrupt ICC networks, and alter smooth muscle responsiveness, ultimately leading to slow transit and motility dysfunction. These processes are interconnected and form a bidirectional regulatory network linking microbiota, immune responses, barrier integrity, and neuromuscular function, thereby sustaining constipation progression.

Although an increasing number of studies have explored the relationships among gut microbiota, immune regulation, and intestinal motility, most existing reviews focus on individual components of this system. A comprehensive synthesis of the integrative mechanisms by which microbiota–immune interactions regulate intestinal motility in constipation remains limited. Therefore, this review focuses on constipation-associated alterations in gut microecology and immune homeostasis, systematically summarizing the potential mechanisms through which microbiota–immune interactions influence intestinal motility. In addition, the dynamic characteristics of these interactions are discussed in the context of disease progression. Furthermore, recent advances in clinical studies on microbiota-targeted interventions for constipation are summarized, with the aim of providing insights into the mechanisms of constipation and informing the optimization of immune-related therapeutic strategies. To conceptually integrate the overall role of microbiota–immune interactions in constipation, we propose a microbiota–immune–motility regulatory axis model, as illustrated in Figure 1.

## 2 Alterations in gut microecology and immune homeostasis associated with constipation

In recent years, advances in high-throughput sequencing and metabolomics have enabled a more systematic characterization of gut microbial alterations associated with constipation. Compared with healthy individuals, several studies have reported reduced microbial diversity and community restructuring in patients with constipation (Zhou et al., 2024). However, the directionality of specific taxonomic changes remains inconsistent, likely reflecting differences in participant age, constipation phenotype, dietary patterns, medication use, as well as sample collection and analytical methodologies (Song et al., 2025; Hu et al., 2025). Rather than focusing solely on compositional shifts, current research increasingly interprets microbiota alterations in terms of functional metabolic reprogramming, particularly involving short-chain fatty acid (SCFA)-related pathways (Zou et al., 2024). SCFAs play a critical role in maintaining epithelial energy metabolism and local microenvironmental stability; reduced SCFA production may compromise mucosal barrier integrity and luminal homeostasis (Liu S. H. et al., 2025). In addition, alterations in bile acid metabolism, mucus utilization, and gas-producing microbial populations may further influence the physicochemical properties of intestinal contents and the local microenvironment (Xia et al., 2025; Zha X. et al., 2023). Overall, constipation-associated microbiota alterations exhibit marked heterogeneity, with features that may dynamically evolve across disease stages and subtypes (Bai et al., 2026; Ma et al., 2025). Notably, most existing studies are based on cross-sectional or small-sample designs, and variations in sequencing platforms and analytical strategies further limit the ability to establish causal relationships between microbiota changes and constipation.

Beyond microbial alterations, the intestinal mucosal immune milieu also undergoes corresponding changes under constipated conditions. Under physiological circumstances, the intestinal immune system maintains a dynamic equilibrium (immune homeostasis), preserving tolerance to commensal microbes while retaining the capacity to respond to harmful stimuli; this balance depends on coordinated regulation among the mucosal barrier, immune cell function, and microbial signals (Ahrend et al., 2025; Wang Q. et al., 2025). In the context of constipation, some studies have observed alterations in immune cell functional states and inflammatory mediator expression (Wu et al., 2026), suggesting a potential shift in immune homeostasis (Li H. et al., 2025). Changes in microbial composition and their metabolites can modulate immune cell activation, cytokine secretion, and the expression of barrier-associated molecules, thereby shaping the immune microenvironment (Jin et al., 2025; Liu X. X. et al., 2025). Conversely, immune responses can in turn influence microbial composition through mechanisms such as antimicrobial peptides, immune exclusion, and mucosal factors (Peña-Juárez et al., 2024). Taken together, microbial dysbiosis and immune dysregulation appear to evolve in parallel and interact dynamically during the course of constipation.

It should be noted that most existing evidence is derived from associative studies, and the direction of causality remains unclear. On the one hand, alterations in the microbiota and their metabolic functions may contribute to the regulation of intestinal motility by modulating immune status, barrier integrity, and enteric neural activity. On the other hand, reduced intestinal motility and prolonged luminal transit time may, in turn, reshape the intestinal microenvironment, thereby influencing microbial composition and metabolic profiles. In addition, factors such as diet, medication use, and host baseline characteristics may act as important confounders. Consequently, the microbiota alterations observed in constipation may represent both contributors to disease pathogenesis and secondary phenomena, and their precise roles and modes of action require further clarification through longitudinal studies and interventional trials.

## 3 Microbiota–immune interactions and their mechanisms in the regulation of intestinal motility

### 3.1 Molecular mechanisms by which the gut microbiota influences the immune system

**Figure 2:**
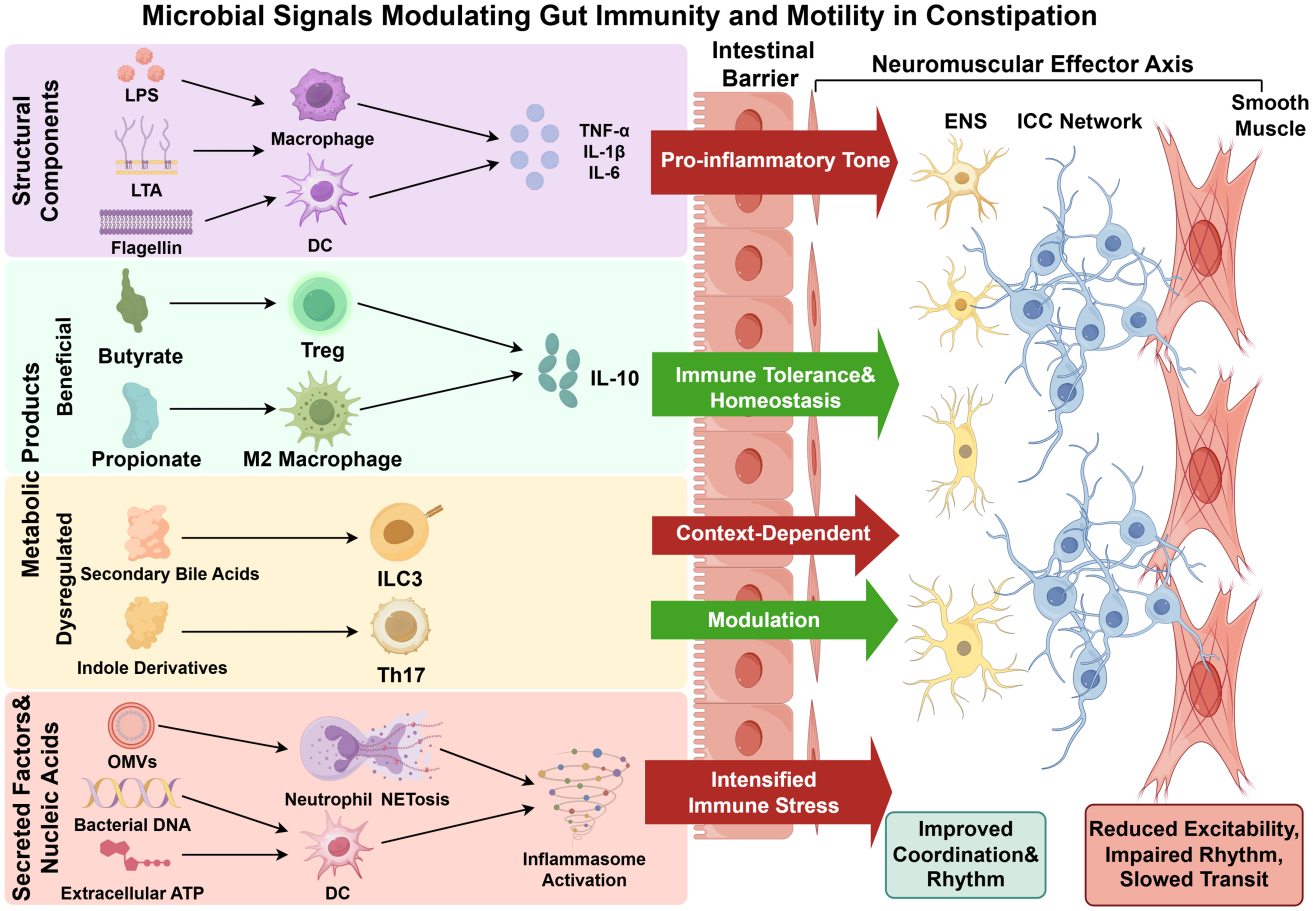
Microbial signals modulating gut immunity and intestinal motility. Microbiota-derived signals regulate intestinal immunity and motility through multiple molecular pathways. Structural components activate innate immune cells, including macrophages and dendritic cells, leading to pro-inflammatory cytokine production and increased inflammatory tone. Beneficial metabolic products, particularly SCFAs such as butyrate and propionate, promote regulatory immune responses by inducing Treg cells and M2 macrophages, supporting immune tolerance and mucosal homeostasis. Dysregulated metabolites, including secondary bile acids and indole derivatives, exert context-dependent effects on immune cell subsets, contributing to immune modulation under pathological conditions. In addition, secreted microbial factors and nucleic acid–related molecules can amplify immune responses through mechanisms such as NETosis and inflammasome activation, leading to intensified immune stress. These immune-mediated signals act on the intestinal barrier and further influence the neuromuscular effector axis, including the ENS, ICC, and smooth muscle cells, thereby modulating coordination, rhythmicity, and contractility of intestinal motility.

The gut microbiota continuously interact with the intestinal immune system through their structural components, metabolites, and secreted or released molecules, thereby shaping the local immune microenvironment (Liu R. et al., 2024; Zheng et al., 2023). To systematically present these pathways, Table 1 summarizes microbial signals, their target cells, and the corresponding immune effects according to different sources of origin. It should be noted that the mechanisms included in the table are derived from studies at various levels of evidence, with most based on animal and *in vitro* models, while direct evidence in humans remains relatively limited. Overall, these microbial signals collectively modulate immune responses to establish an immune context that influences enteric neuronal and smooth muscle function; the integrated mechanisms are illustrated in Figure 2.

**Table 1:** Microbial signals involved in microbiota–immune interactions and their potential effects on intestinal motility.

Category	Microbial molecule	Target cells	Key pathway	Immunoregulatory effects	Effects on intestinal motility	References
Structural component	Lipopolysaccharide	Macrophages; dendritic cells	TLR4 → MyD88 → NF-κB activation	Induction of low-level TNF-α, IL-1β, and IL-6 release; elevation of local inflammatory tone	Decreased ENS excitability; increased peristaltic reflex threshold; slowed neural transmission	Charoensaensuk et al. (2024), Luo et al. (2025)
Structural component	Lipoteichoic acid	Macrophages	TLR2 → NF-κB/MAPK signaling	Modulation of innate immune activation threshold and cytokine profile	Altered smooth muscle responsiveness to neural stimulation; reduced or unstable contractile strength	Xu et al. (2025), Li R. et al. (2024)
Structural component	Flagellin	Dendritic cells; T cells	TLR5 → NF-κB/IL-23 axis → Th17 polarization	Promotion of pro-inflammatory immune polarization (e.g., Th17-associated responses); alteration of cytokine profiles	Altered ENS–immune interface signaling; reduced stability of peristaltic rhythm	Seo and Lim (2025), Vijay-Kumar et al. (2023)
Structural component	Peptidoglycan fragments	Epithelial-associated immune cells; macrophages	NOD1/NOD2 → RIP2 → NF-κB activation	Upregulation of antimicrobial peptides and basal inflammatory mediator expression	Increased background signaling in mucosal–neural reflex pathways; reduced reflex efficiency	Spielbauer et al. (2024), Fioriti et al. (2024)
Structural-associated factor	Bacterial adhesins	Epithelial cells; macrophages	β-catenin signaling/integrin-mediated activation	Promotion of pro-inflammatory macrophage polarization with barrier stress and immune recruitment	Elevated mucosal sensory threshold; weakened initiation of peristaltic reflexes	Yang H. et al. (2025), Smith et al. (2023)
Metabolite	Butyrate	Macrophages; dendritic cells; Treg cells	HDAC inhibition; FFAR2/3 activation → Treg induction	Promotion of M2-like macrophage polarization and tolerogenic DC phenotype; enhancement of Treg responses; suppression of excessive inflammation	Support of ICC pacemaker activity; improved smooth muscle coordination; stabilization of propulsive motility	Yang et al. (2026), Wang et al. (2023a)
Metabolite	Propionate	Treg cells; macrophages	FFAR2/FFAR3 signaling → immune tolerance pathways	Enhancement of Treg differentiation and suppression of excessive inflammatory responses	Stabilization of ENS excitatory–inhibitory balance; improved signal transmission efficiency	Bai et al. (2023), Tang et al. (2023)
Metabolite	Acetate	Neutrophils; ILC3	FFAR2 activation → context-dependent immune signaling	Context-dependent immune modulation, including enhanced chemotaxis or immune tolerance regulation	Modulation of ENS rhythmic activity; motility influenced by inflammatory background	Lee et al. (2023), Wang et al. (2023b)
Metabolite	Branched-chain SCFAs	Macrophages; neutrophils	mTOR/chemotaxis-related signaling modulation	Modulation of immune cell chemotaxis and inflammatory tone	Altered local reflex gain and subtle fluctuations in motility output	Alzubi and Monk (2024), Gao et al. (2025)
Metabolite	Secondary bile acids	Macrophages; dendritic cells; T cells	FXR / TGR5 signaling → IL-10 axis modulation	Upregulation of IL-10-associated regulatory responses and modulation of Th17/Treg balance	Altered smooth muscle response threshold; changes in propagation wave amplitude and frequency	Ding S. Q. et al. (2025), Zheng et al. (2025)
Metabolite	Tryptophan derivatives	ILCs; dendritic cells; epithelial immune cells	AhR activation → IL-22/barrier repair signaling	Maintenance of mucosal immune homeostasis and promotion of barrier-repair phenotypes	Lowered mucosal–neural reflex threshold; improved motility signal transmission	Pimentel et al. (2024), Wang X. et al. (2025)
Metabolite	Neuroactive small molecules	Macrophages; ENS-associated cells	5-HT signaling/GABAergic pathways	Bias toward inhibitory immune regulation and reduction of local inflammatory tone	Reduced ENS excitability and suppression of motility activity	Yao et al. (2025), Tarasiuk-Zawadzka and Fichna (2025)
Secreted factor	Outer membrane vesicles (OMVs)	Macrophages; dendritic cells	TLR-mediated signaling; antigen delivery pathways	Delivery of multiple immunostimulatory signals and amplification of local immune responses	Disruption of ENS–ICC–smooth muscle coupling; reduced coordination of intestinal motility	Wang Y. et al. (2025)
Secreted factor	Bacterial secreted proteins	Epithelial cells; immune cells	NF-κB/epithelial stress signaling	Induction of epithelial stress and immune cell recruitment with maintenance of low-grade inflammation	Increased mucosal stimulation threshold and reduced reflex peristalsis efficiency	Ye and Cai (2025), Alcaino et al. (2025)
Secreted factor	Extracellular ATP	Macrophages; DCs; neutrophils	P2X7 receptor → inflammasome activation	Promotion of inflammatory mediator release and immune cell recruitment	Increased background signaling in reflex pathways; unstable motility patterns	Yuan et al. (2025)
Nucleic acid	Bacterial DNA	Dendritic cells; macrophages	TLR9 → type I interferon signaling	Induction of interferon-like immune responses and cytokine production	Increased ENS microenvironmental stress; reduced reflex efficiency	Feng et al. (2024), Gong et al. (2024)
Nucleic acid	Bacterial RNA	Epithelial cells; dendritic cells	TLR7/8 → antiviral-like immune activation	Activation of antiviral-like immune responses and disruption of epithelial immune homeostasis	Possible disturbance of ICC rhythmic activity; unstable contraction patterns	Kim et al. (2023), Jia et al. (2021)
Nucleic acid	Extracellular DNA	Neutrophils; macrophages	cGAS–STING pathway activation	Amplification of immune activation and maintenance of inflammatory tension	Increased intestinal wall stress and reduced stability of motility regulation	Mishra et al. (2025), Zhao et al. (2025)

#### 3.1.1 Immune regulation mediated by microbial structural components

Microbial structural components, such as lipopolysaccharide (LPS), lipoteichoic acid, and flagellin, can activate immune signaling pathways via pattern recognition receptors, thereby shaping the local intestinal immune environment (Pang et al., 2025; Jaishankar et al., 2025). In the context of constipation, shifts in the relative abundance of Gram-negative bacteria may be accompanied by alterations in LPS-associated signaling load, which can drive mucosal macrophages toward a pro-inflammatory phenotype through the TLR4/NF-κB pathway. This process promotes the sustained release of inflammatory mediators, including TNF-α and IL-1β, thereby maintaining a state of low-grade immune activation (De Chiara et al., 2025; Furnari et al., 2025). Animal studies suggest that modulation of LPS-related signaling can influence inflammatory mediator levels in parallel with changes in intestinal motility indices, indicating a potential role in motility regulation via immune-mediated mechanisms (Li Z. et al., 2024). However, in human studies, the relationship between LPS levels and constipation severity remains inconsistent, suggesting that its role may be better understood as modulation of the immune milieu rather than a direct determinant. In addition, flagellin can influence dendritic cell and T cell function and modulate cytokine profiles, while bacterial adhesins and surface polysaccharides may contribute to mucosal immune homeostasis by promoting either pro-inflammatory or repair-associated phenotypic transitions, respectively (Deb et al., 2024; Cheng et al., 2025).

#### 3.1.2 Immune regulation mediated by microbial metabolites

The gut microbiota can indirectly interact with immune cells through the production of a wide range of metabolites, thereby playing a critical role in maintaining immune homeostasis and regulating immune function (Zhang X. et al., 2025). Short-chain fatty acids (SCFAs), secondary bile acids, tryptophan-derived metabolites, and certain neuroactive small molecules can act on diverse immune cell populations to modulate local immune tone, and subsequently influence the enteric nervous system and smooth muscle function (Zinkow et al., 2024; Çelik et al., 2026). Notably, most of the current evidence is derived from animal models and *in vitro* studies, and mechanistic validation in human populations remains relatively limited.

Different types of SCFAs exert distinct immunomodulatory effects (Liu et al., 2023). Butyrate and propionate have been shown in multiple studies to promote regulatory T cell differentiation and suppress excessive inflammatory responses, thereby supporting immune tolerance and reducing inflammation-induced interference with neural regulation (Li Q. et al., 2025; Liu Z. et al., 2025). In contrast, the effects of acetate appear to be context-dependent, exhibiting either pro- or anti-inflammatory properties under different conditions (Lee J. et al., 2025). Overall, SCFAs contribute to the regulation of intestinal neuro–immune–motility interactions by modulating the magnitude and balance of immune responses, although their specific roles in constipation remain heterogeneous and not fully consistent. In addition, secondary bile acids and tryptophan-derived metabolites can modulate immune cell functional states, contributing to the balance between inflammation and immune tolerance, and regulate peristaltic reflexes by influencing signal integration across the epithelial–immune–neural axis (Baldi et al., 2026; Lin et al., 2026).

#### 3.1.3 Immunological effects of microbial secreted factors and nucleic acid–related molecules

Beyond structural components and metabolites, the gut microbiota can also exert sustained effects on the host immune system through the release of diverse secreted factors and nucleic acid–associated molecules (Almansour et al., 2025). These signals typically do not directly elicit strong inflammatory responses; rather, they contribute to immune microenvironmental regulation by modulating activation thresholds and amplifying signaling cascades (Dawson et al., 2025). Outer membrane vesicles (OMVs), as representative structures, can carry a variety of microbe-derived molecules and be taken up by immune cells, thereby enhancing local immune responses and promoting immune cell recruitment. In parallel, bacterial secreted proteins may indirectly facilitate immune activation by inducing epithelial stress responses (Miao et al., 2025). In addition, extracellular ATP, as well as bacterial DNA and RNA, can function as immunostimulatory signals and participate in the amplification of inflammatory pathways (Baca and Marraffini, 2025). In animal models, these signals have been associated with sustained immune activation and alterations in barrier function (Lu et al., 2026). However, in human studies, their dynamic patterns and their relationship with intestinal motility disturbances remain insufficiently characterized. Overall, these molecules are more likely to influence enteric neural regulation and the stability of peristaltic reflexes by enhancing immune signal amplification and elevating local immune stress levels (Wang H. et al., 2025).

### 3.2 Reciprocal regulation of gut microbiota composition and spatial distribution by the immune system

The ability of the immune system to discriminate between commensal microbes and potentially harmful organisms is fundamental to maintaining gut microbial stability (Tay et al., 2025). Upon recognition of commensal microbiota, immune responses are typically downregulated to preserve immune tolerance; in contrast, when aberrant microbial signals are detected or barrier integrity is compromised, immune responses are activated to exert selective clearance pressure on specific microbial populations, thereby regulating their composition and spatial distribution (Shao et al., 2023; Ajith and Anita, 2025). In this process, innate immune cells play a central role. Macrophages can selectively influence microbial communities by modulating the production of effector molecules such as nitric oxide (Tian et al., 2025), while mononuclear phagocytes and neutrophils, once activated within specific microenvironments, can release a range of reactive mediators that limit microbial overgrowth and translocation (Weppner et al., 2025; Fu et al., 2025b). Animal studies have shown that disruption of macrophage or neutrophil function leads to alterations in microbial composition and stability, whereas modulation of related immune pathways can help restore microbial balance, underscoring the critical role of innate immunity in maintaining microbial homeostasis (Cui et al., 2025).

In addition, changes in the expression of immune receptors can influence the host’s capacity to sense microbial signals, thereby further shaping microbial community structure (Liu X. et al., 2024). It should be noted, however, that the bidirectional interplay between immune regulation and microbial dynamics is highly complex, and the spatial organization of the microbiota remains difficult to precisely assess, leaving the underlying mechanisms incompletely understood. In the context of constipation, reduced intestinal motility and prolonged luminal transit time may further amplify these regulatory processes, although the detailed mechanisms require further investigation.

### 3.3 Interaction mechanisms among the gut microbiota, immune system, and intestinal motility

The interplay among the microbiota, the immune system, and intestinal motility is not linear, but rather constitutes a dynamically regulated feedback loop (Yang S. et al., 2025). In the context of chronic constipation, this regulatory network may progressively amplify from an initial imbalance. Reduced intestinal motility prolongs luminal transit time, thereby increasing mucosal exposure to microbe-associated signals and altering the local immune environment (Iyer et al., 2023). Changes in immune status, in turn, selectively shape microbial composition and metabolic profiles (Woo and Ki, 2025), while the altered metabolic milieu further suppresses propulsive motor function.

Concurrently, sustained low-grade immune activation can influence enteric neuronal excitability and neurotransmitter release through inflammatory mediators, and disrupt the rhythmic signaling and electrical coupling stability of interstitial cells of Cajal (Lee et al., 2026; Girardi et al., 2023). Impairment of barrier function and the translocation of microbiota toward the mucosal surface further enhance immune exposure to microbial signals (Schreiber et al., 2024). Through these multi-level interactions, alterations in microbial structure, immune status, and intestinal motility reinforce one another, ultimately forming a self-sustaining positive feedback loop that maintains the disease state. However, current evidence is largely derived from isolated experimental studies, and systematic validation of the dominant nodes and causal relationships within this feedback loop across different constipation phenotypes remains lacking.

## 4 Mechanistic evolution of immune–microbiota interactions in the development and progression of constipation

From a disease course perspective, the onset and progression of constipation are not driven by abnormalities in a single component, but rather reflect a dynamic process involving the gradual imbalance and interaction of multiple systems, including the gut microbiota, mucosal immunity, barrier function, and intestinal motility regulation (Ihara et al., 2025). In the early stage, the condition is primarily characterized by increased microbe-associated stimulation and a mild shift in baseline immune status. This is followed by fluctuations in mucosal barrier function, which facilitate greater translocation of microbial signals into the mucosal compartment (Chen et al., 2024).

**Figure 3:**
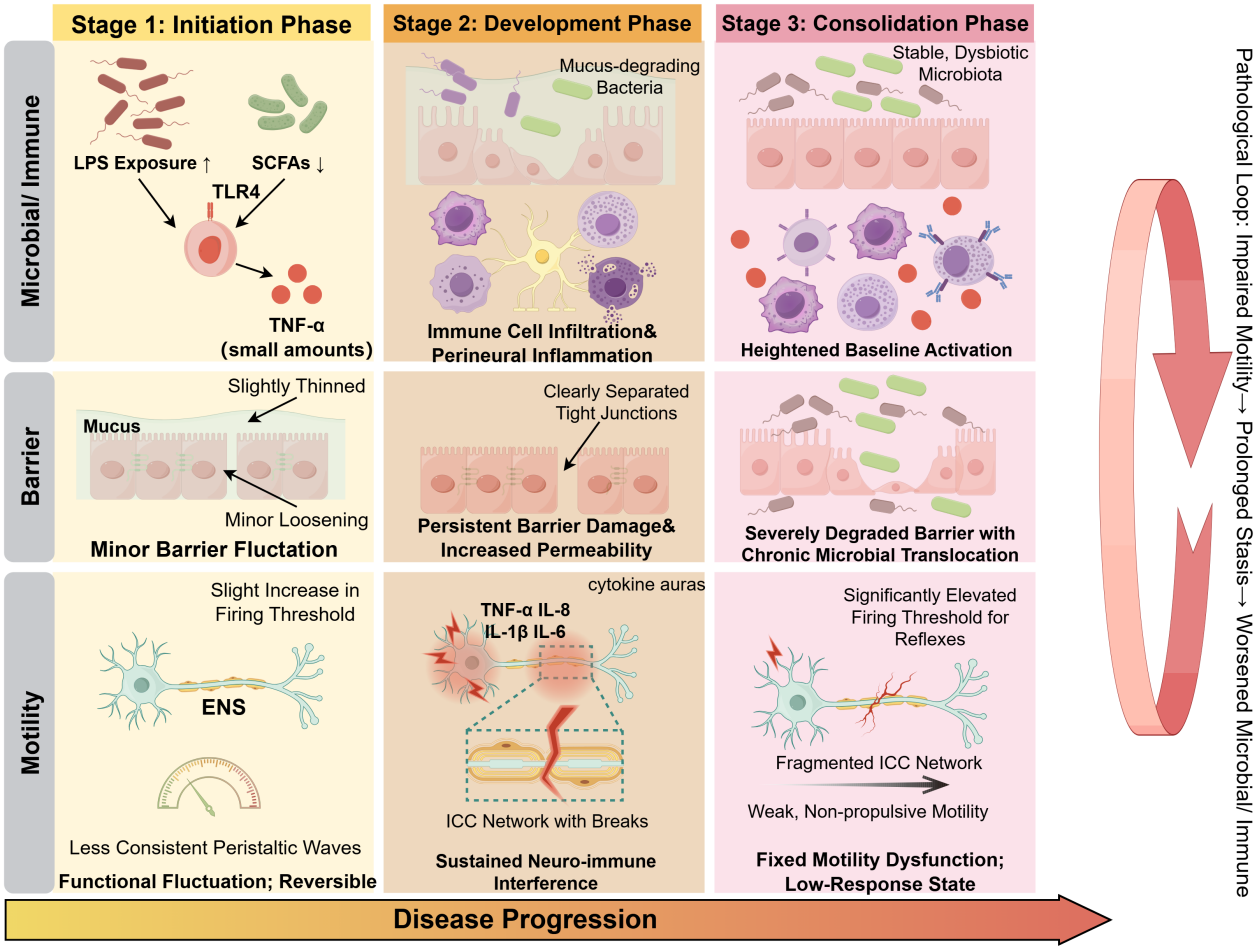
Proposed progression model of microbiota–immune dysregulation in constipation. Constipation develops through a staged process involving coordinated alterations in gut microbiota, mucosal immune responses, epithelial barrier integrity, and neuromuscular regulation. In the initiation phase, increased microbial-derived signals and mild immune activation occur with relatively preserved barrier function. During the development phase, sustained immune activation and barrier dysfunction lead to increased epithelial permeability and enhanced microbial–host interactions, accompanied by neuro-immune interference. In the consolidation phase, persistent dysbiosis, chronic immune activation, and structural barrier damage contribute to impaired enteric nervous system function, disruption of ICC networks, and weakened smooth muscle responses, resulting in stable motility dysfunction. Importantly, these processes are interconnected and form a multi-level positive feedback loop linking impaired motility, microbial dysbiosis, barrier disruption, and immune activation, thereby sustaining disease progression.

As the disease progresses, immune responses and their mediators extend deeper into the intestinal wall and begin to affect neural regulatory structures, leading to a transition from reversible fluctuations in motility to persistent functional impairment (Hou and Artis, 2025). Based on current evidence, this process can be broadly categorized into an initiation stage, a progression stage, and a consolidation stage of motility dysfunction. Throughout these stages, the gut microbiota, immune responses, barrier integrity, and motility regulation interact across multiple levels to form a positive feedback loop, thereby driving the sustained progression of the disease (Figure 3).

### 4.1 Interactions between immunity and the gut microbiota in the initiation stage of constipation

#### 4.1.1 Low-grade immune activation and impaired immune regulation

In the initiation stage of constipation, early alterations are typically characterized by increased exposure to microbe-associated structural components and shifts in metabolic profiles (Zhang et al., 2024). In models of slow-transit constipation, an increased proportion of Gram-negative bacteria has been observed, accompanied by elevated exposure to lipopolysaccharide (LPS) (Iancu et al., 2023). Persistent LPS stimulation can activate mucosal macrophages and dendritic cells via the TLR4/NF-κB pathway, leading to sustained low-level expression of inflammatory mediators such as TNF-α, IL-1β, and IL-6, thereby establishing a state of chronic low-grade immune activation (Sun et al., 2023). During this process, macrophage phenotypes and cytokine profiles undergo shifts, while certain opportunistic commensals may induce the upregulation of regulatory signals such as IL-10 and TGF-β. Although these responses help limit excessive inflammation, they may also reduce the efficiency of clearing persistent microbial stimuli (Zha C. et al., 2023; Bao et al., 2024).

Sustained alterations in immune status can further impact the enteric neuro–muscular regulatory system. Transcriptomic and experimental studies suggest that an inflammatory milieu can modulate neuronal excitability and neurotransmitter release within the myenteric plexus, disrupt the rhythmic signaling and electrical coupling stability of interstitial cells of Cajal, and alter smooth muscle responsiveness to excitatory and inhibitory neural inputs, manifesting as delayed contraction initiation and unstable rhythmicity (Hamnett et al., 2025; Varela et al., 2025; Faggin et al., 2025). Thus, microbiota-driven low-grade immune activation may continuously interfere with neural and smooth muscle regulation through pathways such as TLR4/NF-κB and their downstream signaling cascades, thereby contributing to the development of slowed intestinal transit. However, these mechanisms are largely supported by animal studies, and their dominant roles and causal relationships in human populations remain to be further elucidated.

#### 4.1.2 Alterations in barrier function

Imbalance in the gut microbiota and immune regulation can compromise the structural and functional stability of the mucosal barrier, facilitating the accumulation of microbial populations with adhesive or mucus-utilizing capacities at the epithelial surface, thereby increasing the likelihood of contact between microbe-associated molecules and the intestinal wall (Duan et al., 2023). In constipation models, impaired goblet cell function and reduced mucus secretion are often observed, accompanied by thinning of the mucus layer, which allows bacteria to more readily approach the epithelial surface (Zhou et al., 2023; Yoo and Oh, 2023). Concurrently, the expression of tight junction proteins, including occludin, claudins, and ZO-1, is downregulated, leading to increased epithelial permeability and facilitating the translocation of lipopolysaccharide, peptidoglycan fragments, and certain metabolites into the mucosal compartment (Lacy and Cangemi, 2024).

Barrier dysfunction may also interfere with epithelial-associated neural reflex regulation. Enterochromaffin cells, which are sensitive to microbial metabolites and mechanical stretch, can exhibit functional alterations that disrupt the synthesis and release patterns of 5-hydroxytryptamine (5-HT), thereby reducing the efficiency of mucosal reflex initiation and impairing the coordination of propulsive contractions (Moretti et al., 2025). In addition, changes in epithelial ion transport and water reabsorption may decrease fecal water content and promote stool hardening, increasing resistance to luminal propulsion and further exacerbating delayed transit (Huang et al., 2025). During this process, the enrichment of microbiota at the mucosal surface, along with adjustments in IgA coating and antimicrobial peptide expression, may sustain low-level immune stimulation and establish a localized positive feedback microenvironment (Ding G. et al., 2025).

#### 4.1.3 Intestinal motility dysfunction

Against a background of mild immune deviation and compromised mucosal barrier function, sustained exposure of the intestinal wall to microbe-associated signals can progressively disrupt the homeostasis of motility regulation (Campbell et al., 2023). Evidence suggests that alterations in SCFA levels or the accumulation of other metabolites may increase the response threshold of myenteric plexus neurons to mechanical stretch and luminal chemical stimuli, thereby reducing signal transmission efficiency and decreasing both the frequency and strength of propulsive contractions (Thomasi et al., 2025).

Intestinal motility dysfunction reflects a coordinated imbalance across multiple regulatory levels, including neural control, rhythm generation, and effector execution. Interstitial cells of Cajal (ICC), which serve as pacemakers and propagation networks, are particularly sensitive to inflammatory mediators and epithelial stress. Reductions in ICC number or network integrity can impair the speed and synchrony of rhythmic signal propagation, making it difficult to maintain stable propulsive contraction patterns (Liu M. et al., 2025; Du et al., 2025; Baek et al., 2025). Meanwhile, alterations in neurotransmitter release dynamics and local metabolic disturbances can affect smooth muscle responsiveness to excitatory cholinergic signals and inhibitory inputs such as nitric oxide, manifesting as delayed contraction onset, reduced amplitude, and irregular rhythmicity (Li L. et al., 2024).

Through the combined effects of these mechanisms, coordinated motility regulation can be disrupted even in the absence of overt histological damage, resulting in reduced propulsive activity, discontinuous rhythmic patterns, and slowed intestinal transit. However, most of the current evidence is derived from animal studies, and the magnitude of these effects in humans, as well as the dominant mechanisms across different constipation subtypes, remain to be further clarified.

### 4.2 Amplification of microbiota–immune interactions during the progression stage of constipation

#### 4.2.1 Persistent damage to the mucosal barrier

As constipation progresses, mucosal barrier abnormalities evolve from early functional fluctuations into sustained structural and functional impairment, characterized by reduced reparative capacity and persistently increased permeability. The affected regions extend from the mucus layer to epithelial junctional complexes (Zhang Y. et al., 2025; Jin et al., 2024). Impaired goblet cell secretion and thinning of the mucus layer weaken mucosal separation capacity, while continued downregulation of tight junction proteins, accompanied by increased permeability, indicates stable disruption of barrier integrity (Leoncini et al., 2024).

Persistent barrier damage not only increases the entry of microbe-associated molecules into the mucosa but also prolongs their contact with the intestinal wall, shifting immune stimulation from intermittent to continuous. Meanwhile, alterations in epithelial ion transport and water reabsorption lead to luminal dehydration and stool hardening, increasing resistance to propulsion. This, in turn, exacerbates mechanical stress on the mucosa and delays barrier repair, promoting the transition from focal abnormalities to stable structural defects (Tian et al., 2024; Sandle and Rajendran, 2025).

In the context of reduced intestinal motility, prolonged transit time facilitates the accumulation of microbiota at the mucosal surface. Certain microbial populations establish relatively stable colonization through adhesion or mucus utilization, continuously consuming mucus and sustaining immune stimulation. Concurrently, alterations in IgA coating and antimicrobial peptide expression further promote microbial localization toward the mucosal region, allowing microbial signals to act persistently on submucosal tissues (Bai et al., 2024). Collectively, these processes establish a reinforcing positive feedback loop involving barrier impairment, immune stimulation, and microbial enrichment.

#### 4.2.2 Involvement of neural regulation

During the progression stage, immune cells and their mediators may extend deeper into the intestinal wall and increasingly localize near key neural regulatory structures, such as the myenteric plexus (Yang W. et al., 2025). This process is typically not characterized by overt, high-grade inflammation; rather, it reflects sustained, low-level immune activity that spatially approaches neural structures, thereby exerting chronic interference with local neural regulation (Tuncel et al., 2025). Histological studies have demonstrated an increased density of immune cells surrounding neural plexuses, accompanied by altered spatial relationships between immune cells, nerve fibers, and ganglia (Stoyanova et al., 2025). In models of chronic constipation, macrophages and mast cells are often enriched in the vicinity of neural plexuses, along with elevated local levels of inflammatory mediators (Zenatri et al., 2025; Lobo et al., 2023).

The reduced spatial distance between immune cells and neural structures facilitates the formation of higher effective concentrations of inflammatory mediators in the local microenvironment, thereby amplifying their disruptive effects on neural regulation. At the molecular and functional levels, mediators such as TNF-α, IL-1β, and IL-6 can alter neuronal membrane potential, ion channel expression, and neurotransmitter release, leading to reduced efficiency of neural signal transmission and impaired precision of reflex control (Seguella et al., 2026; Haider et al., 2025). In parallel, the homeostatic maintenance and reparative support functions of enteric glial cells may also be affected by persistent immune stimulation. Altered expression of neurotrophic factors can diminish the compensatory and regenerative capacity of the neural network (Gonzales and Gulbransen, 2025; Mao and Shen, 2024).

#### 4.2.3 Stabilization of intestinal motility dysfunction

With further disease progression, the persistent presence of mucosal barrier damage and the sustained extension of immune responses into deeper layers of the intestinal wall drive a transition from early reversible fluctuations to stable functional abnormalities in intestinal regulation (Feng et al., 2023). At this stage, the pathology is no longer characterized by transient imbalance within a single component; rather, long-term alterations in microbial composition, immune activity, and neural regulation occur concurrently and reinforce one another, collectively sustaining impaired intestinal motility.

At the microbial level, reduced motility prolongs luminal transit time, creating a more favorable niche for microbial populations with higher tolerance or adhesive capacity. Their distribution and metabolic profiles gradually stabilize (Ma et al., 2023; Panarese, 2024). These changes may not necessarily manifest as a marked decline in microbial diversity, but their metabolic outputs are more likely to sustain mucosal stimulation and low-grade immune activation, thereby hindering the recovery of motility function (Parkar et al., 2023).

At the immune level, the mucosa typically exhibits a state of persistent low-grade activation, characterized by a modest increase in immune cell abundance and elevated baseline levels of inflammatory mediators, along with heightened responsiveness to microbial and metabolic signals (Yang et al., 2023; Zhou et al., 2025). This condition may induce amplified functional responses, leading to repeated disruption of neural regulation and reducing the system’s capacity to return to homeostasis. Meanwhile, chronic aberrant stimulation can progressively increase the activation threshold of motility reflexes (Zhao et al., 2024), such that mechanical or chemical stimuli of equivalent intensity become insufficient to effectively initiate propulsive waves. Consequently, both neural transmission efficiency and smooth muscle contractile responsiveness decline.

Overall, throughout the course of constipation, microbial composition and metabolic function, barrier integrity, and immune responses undergo a dynamic evolution from early deviations to sustained amplification. The early stage is primarily characterized by increased microbial-associated molecular load and shifts in baseline immune status, with barrier fluctuations facilitating greater access of these signals to the mucosal environment. As the disease progresses, persistent barrier impairment and the spatial extension of immune activity into deeper intestinal layers increase the susceptibility of neural regulatory structures to disruption, leading to a gradual transition from reversible motility disturbances to stable dysfunction. With prolonged disease duration, interactions among microbial metabolic profiles, immune responses, and neural regulation become mutually reinforcing, maintaining intestinal motility in a low-responsiveness state. However, inconsistencies remain across studies regarding the directionality of microbiota alterations and their associations with clinical phenotypes. The dominant roles and causal relationships of these factors across different constipation subtypes have yet to be clearly defined.

It should be emphasized that the proposed “microbiota–immune–motility” interaction axis presented here is largely derived from the integration of evidence across multiple levels of research. While certain mechanistic components have been supported by animal and *in vitro* studies—for example, the regulation of immune responses via the TLR4/NF-κB pathway by microbe-associated molecules, and the effects of inflammatory mediators on enteric nervous system function—direct evidence supporting the full causal chain linking microbiota alterations to immune modulation and subsequently to motility dysfunction in human populations remains limited. Therefore, this interaction axis should be regarded, to some extent, as an integrative hypothesis based on current evidence. Its dominant pathways and key regulatory nodes across different constipation phenotypes require further validation. Future studies should employ longitudinal designs and integrated multi-dimensional mechanistic assessments to clarify causal relationships among these components and their specific roles in disease onset and progression.

## 5 Clinical advances in microbiota-targeted therapies for constipation

**Figure 4:**
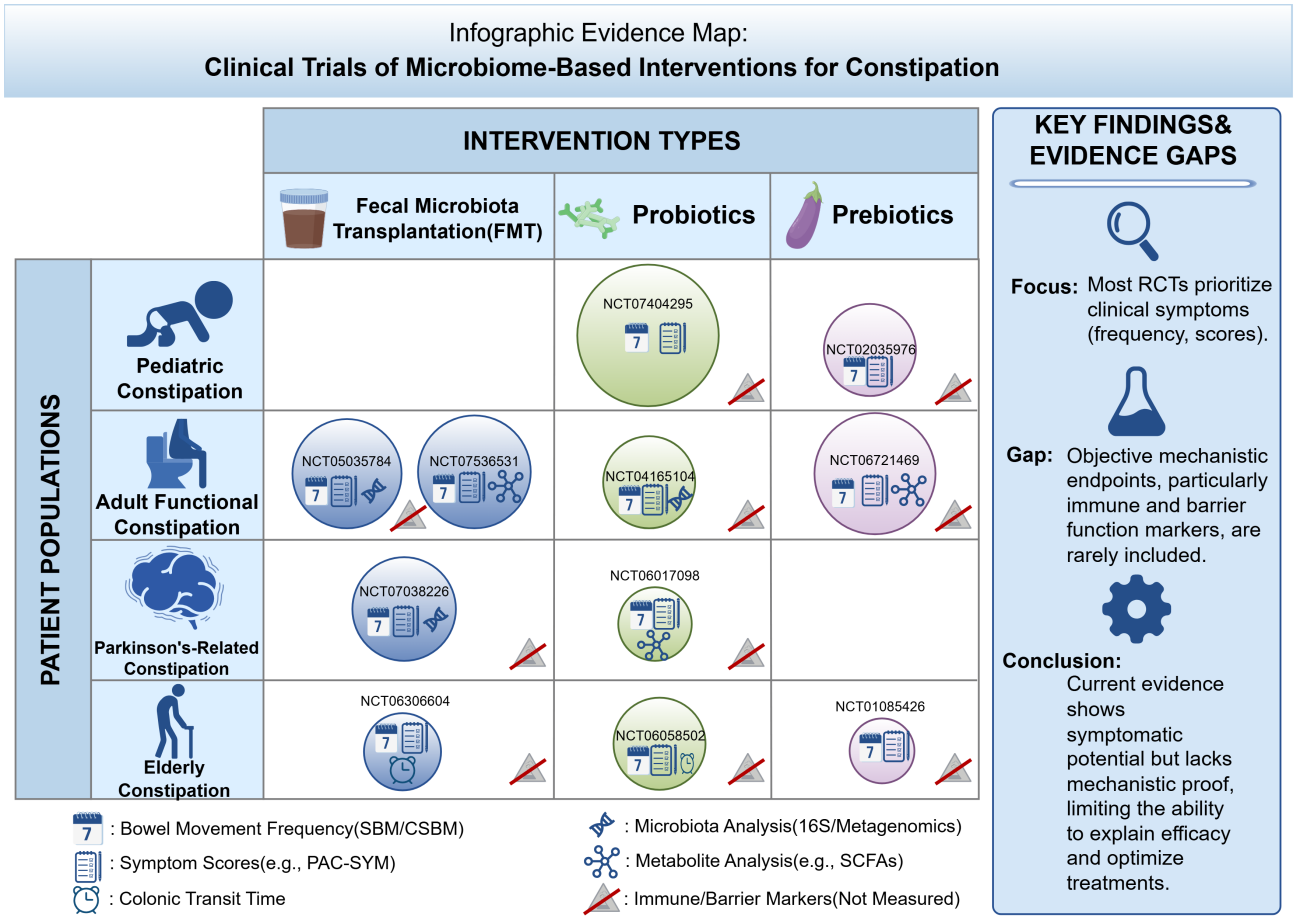
Clinical evidence landscape of microbiome-based interventions for constipation. This figure summarizes clinical trials evaluating microbiota-targeted interventions, including FMT, probiotics, and prebiotics, across different patient populations. Each circle represents an individual study, with symbols indicating reported clinical outcomes and whether microbiota, metabolite, or immune/barrier-related analyses were performed. Overall, current evidence is unevenly distributed across intervention types and patient populations, with probiotics being the most frequently studied, while FMT and prebiotics remain less extensively investigated in certain groups. Most trials primarily focus on clinical symptom improvement, whereas objective mechanistic endpoints—particularly immune and epithelial barrier markers—are infrequently assessed. These findings highlight substantial evidence gaps in linking microbiota-targeted interventions to underlying biological mechanisms, underscoring the need for more comprehensive and well-designed clinical studies integrating multi-level outcome measures.

Building upon mechanistic studies, interventions targeting the gut microbiota have gradually entered the stage of clinical evaluation for the treatment of constipation. To systematically summarize current clinical research on microbiota-related interventions for constipation, and to illustrate the distribution of different intervention strategies across study populations as well as existing evidence gaps, this review presents an evidence map of microbiota-based interventions (Figure 4).

To evaluate the clinical efficacy of these interventions and to examine whether immune-related indicators were incorporated into outcome assessments, this review also compiled completed controlled studies based on publicly available information from clinical trial registries. The relevant studies are summarized in Table 2. Current clinical interventions primarily include fecal microbiota transplantation (FMT), probiotics, prebiotics, and synbiotics. In most studies, the sample size ranges from several dozen to approximately 100 participants, and the intervention duration is typically 6–8 weeks, indicating that current research remains largely exploratory and short-term in nature. Moreover, substantial heterogeneity exists across studies in terms of strain selection, dosage regimens, and outcome assessment criteria, which further contributes to variability in reported results.

**Table 2:** Results of randomized controlled trials of gut microbiota–targeted interventions for constipation.

Type	NCT number	Sample size	Intervention measures	Disease type	Outcome indicators
FNT	NCT07038226	16	Oral capsule-delivered FMT	Refractory constipation in Parkinson’s disease	Change from baseline in 39-item Parkinson’s disease questionnaire
FNT	NCT05035784	110	Fecal supernatant	Childhood constipation	improvement of spontaneous bowel movements per week
FNT	NCT03308461	22	Fecal microbiota suspension	Constipation	defecation frequency, stool consistency
Probiotics	NCT07152795	90	Probiotic	Chronic constipation in adults	Assessing the improvement of chronic constipation symptoms following probiotic intervention
Probiotics	NCT07002489	102	Nuotelande Shuhuajun study product	Constipation in preschool children	Change of constipation indicators-SCFA level in the fecal samples; Change of constipation indicators—Probiotics—Lactobacillus and Bifidobacterium concentration level in the fecal samples
Probiotics	NCT06879587	84	Probiotic	Functional constipation in adults	Change in colonic transit time from baseline to 8 weeks; Change in patient assessment of constipation symptoms score from baseline to 8 weeks
Prebiotics	NCT02183766	20	Galactooligosaccharide prebiotic	Children constipation	Evidence of constipation improvement after GOS use in children compared to use of placebo
Prebiotics	NCT06381193	80	Prebiotic	Chronic and functional constipation	To evaluate the effect of the dietary supplements on the bowel frequency movements in patients with chronic functional constipation
Prebiotics	NCT05923723	56	Dietary intervention	Constipation in institutionalized older adults	Adequate relief at 6 weeks

### 5.1 FMT

Fecal microbiota transplantation (FMT), as a strategy for whole-community microbial reconstruction, represents one of the most clinically translatable microbiota-based interventions currently available (Hou et al., 2025). Its core principle lies in restoring the structure and functional network of a disrupted host microbiota through the transfer of an intact gut microbial community from healthy donors (Ruan et al., 2025). According to the randomized controlled trials summarized in Table 2, although existing FMT studies are generally limited by small sample sizes, preliminary evidence of efficacy has emerged. For instance, trials (NCT05035784, NCT03308461) have shown that FMT can, to some extent, increase the frequency of spontaneous bowel movements and improve stool consistency. In patients with refractory constipation associated with neurological disorders (NCT07038226), FMT may also contribute to improvements in quality-of-life measures.

Animal studies further suggest that FMT can enhance intestinal barrier function and modulate the distribution of immune cells, thereby supporting the stability of intestinal rhythmic activity (Dai et al., 2025). Following FMT, the expression of barrier-associated proteins is upregulated, inflammatory signaling is attenuated, and the neuro-regulatory environment of the gut is improved (Masi et al., 2026). In addition, metabolomic analyses have demonstrated alterations in SCFAs and bile acid profiles after FMT, which may be linked to the regulation of immune homeostasis and improvements in intestinal motility (Wang et al., 2026). These findings provide new mechanistic insights into the effects of FMT.

Despite these advances, several challenges remain for the clinical application of FMT in constipation. First, heterogeneity in therapeutic outcomes may arise from differences in donor microbiota, transplantation protocols, and the baseline microbial and immune status of recipients. Second, most existing studies focus primarily on short-term symptom relief and lack comprehensive assessments of immune and motility-related parameters, limiting mechanistic interpretation at the clinical level. Finally, the long-term safety of FMT and its sustained impact on immune homeostasis remain insufficiently characterized due to a lack of extended follow-up data.

### 5.2 Probiotic intervention

Probiotic interventions primarily aim to modulate the gut microecological environment through specific microbial strains, thereby influencing mucosal immune status and intestinal motility (Maldonado-Galdeano et al., 2025). Current clinical evidence suggests that probiotics may confer therapeutic benefits in subsets of patients with constipation. Trials (NCT07152795, NCT06879587) have demonstrated that probiotic supplementation can improve bowel movement frequency, stool consistency, and constipation symptom scores, with some studies also reporting a reduction in colonic transit time. In pediatric populations (NCT07002489), probiotic interventions have been associated with changes in fecal SCFA levels and increased abundance of Lactobacillus and Bifidobacterium, indicating potential functional modulation of the gut microbiota.

Mechanistically, specific probiotic strains may regulate immune signaling pathways via pattern recognition receptors, promoting the expression of anti-inflammatory mediators and attenuating low-grade inflammation, thereby providing a more stable regulatory environment for the enteric nervous system (Stierschneider and Wiesner, 2023). Probiotics can also modulate mucosal immune signaling to upregulate IL-10, IL-22, and secretory IgA, facilitating epithelial repair and restoration of tight junction proteins, ultimately improving barrier function (Abbaszadeh et al., 2026). In addition, probiotics may influence macrophage functional states by suppressing pro-inflammatory responses and promoting repair-associated phenotypes, contributing to the maintenance of local immune homeostasis (Filidou et al., 2024).

Furthermore, certain strains may modulate immune cell metabolism and transcriptional regulatory networks, enhancing regulatory T cell–associated responses and stabilizing their functional phenotype, thereby maintaining immune balance under conditions of low-grade inflammation (Kwon et al., 2025). Some studies have reported that *Lactobacillus rhamnosus*, for example, can regulate epithelial signaling pathways, promote epithelial regeneration, and support the establishment of local immune tolerance (Schalich et al., 2023). Moreover, multi-strain probiotic formulations may help restore Th17/Treg balance and reduce inflammation-related signaling, thereby providing a more stable immune environment for the regulation of intestinal motility (Hu et al., 2025).

### 5.3 Prebiotics

In contrast to probiotics, prebiotics primarily exert their effects by altering substrate availability, thereby indirectly modulating microbial composition and metabolic output (Fu et al., 2025a). Clinical studies (NCT02183766, NCT06381193, NCT05923723) have shown that oligosaccharides or dietary interventions can, to some extent, improve bowel movement frequency and symptom relief rates; however, substantial variability exists across studies. This suggests that the efficacy of prebiotics is more likely dependent on the host’s baseline microbiota and metabolic context. Mechanistically, prebiotics promote the production of SCFAs, which may contribute to mucosal immune regulation and the maintenance of barrier function (Feng et al., 2025). In addition, prebiotics may enhance mucosal barrier integrity, thereby reducing the translocation of microbe-associated stimuli into the mucosa and mitigating the impact of low-grade immune activation on intestinal motility (Mo et al., 2024).

It is important to emphasize that the effects of prebiotic interventions are highly dependent on the host’s baseline microbial composition. Only in the presence of functionally relevant microbial populations can substrate availability be translated into meaningful metabolic outputs, which may partially explain the heterogeneity in clinical outcomes (He et al., 2026). Furthermore, dietary patterns, dosage, and duration of intervention may significantly influence therapeutic efficacy. Most current studies focus on short-term outcomes and lack comprehensive mechanistic assessments and long-term follow-up data.

In addition to direct microbiota-targeted interventions, certain non-pharmacological therapies—such as biofeedback, electrical stimulation, and pelvic floor training—have also demonstrated efficacy in the management of constipation. These approaches primarily improve pelvic floor coordination and colonic evacuation efficiency, thereby directly modifying luminal transit time and motility patterns (Geng et al., 2026). Current evidence suggests that prolonged intestinal transit time may promote the accumulation of protein fermentation–related metabolites, whereas shortened transit time may increase the proportion of carbohydrate fermentation, thereby altering SCFA production profiles (Sheng et al., 2023). On this basis, changes in microbial metabolites may further influence mucosal immune status, for example by modulating low-grade inflammation or barrier function (Elinav et al., 2024). However, most clinical studies to date have focused on motility parameters and symptom improvement, with limited concurrent assessment of microbial composition, metabolic outputs, and immune-related markers. Therefore, whether physical therapies can directly modulate the gut microbiota remains to be conclusively demonstrated.

It should be further emphasized that although most existing studies adopt randomized controlled trial (RCT) designs and thus provide a relatively high level of evidence, a certain degree of inconsistency persists across reported outcomes. Some studies have demonstrated that microbiota-targeted interventions can improve bowel movement frequency and symptom scores, whereas others have failed to observe significant benefits. These discrepancies may be closely related to differences in constipation subtypes, baseline microbial composition, dietary habits, and lifestyle factors among study populations. In addition, variability in inclusion criteria and intervention protocols across studies may further affect the comparability of results.

From a study design perspective, many current RCTs are limited by relatively small sample sizes and substantial population heterogeneity, which may compromise the robustness and generalizability of findings. Moreover, most studies primarily rely on clinical symptoms as primary endpoints, while systematic assessments of microbial composition, immune status, and related metabolic parameters remain insufficient. As a result, the relationship between observed clinical effects and the underlying mechanisms remains unclear. Consequently, the effects of microbiota-targeted interventions in constipation appear to be highly individual-dependent, and both their clinical efficacy and mechanistic basis require further clarification through larger-scale studies incorporating multidimensional outcome measures.

Overall, clinical research on microbiota-based interventions in constipation remains at an exploratory stage. Although some studies have reported symptomatic improvements, substantial variability exists across different intervention strategies, and consistent conclusions are lacking. This instability likely reflects, on the one hand, the influence of multiple interacting factors, including microbial composition, host immune status, and dietary patterns, and on the other hand, the lack of standardization in study design, particularly with respect to strain selection, dosing regimens, and outcome measures. Furthermore, the predominant focus on symptom-based outcomes, without concurrent evaluation of immune and motility-related parameters, continues to hinder the clarification of causal relationships within the “microbiota–immune–motility” axis. Future studies should therefore incorporate stratified patient populations and integrate microbiological, immunological, and motility-related metrics to more precisely define the mechanisms and appropriate applications of different intervention strategies.

## 6 Limitations and future perspectives

Based on current evidence, the interplay among the gut microbiota, the immune system, and intestinal motility provides an integrative and conceptually coherent framework for understanding the development and persistence of constipation. Mechanistic links—such as microbiota-derived signals mediating immune regulation and the impact of inflammatory states on enteric neural and motor function—have been supported to some extent by animal and *in vitro* studies. However, the continuous causal chain linking microbial alterations, immune deviation, and motility dysfunction, particularly the dominant pathways and their dynamic evolution across different constipation phenotypes, remains insufficiently validated in human populations.

From a clinical and translational perspective, most existing studies rely primarily on symptom-based outcomes and lack systematic integration of microbiota, immune, and motility-related parameters, resulting in a gap between mechanistic inference and clinical efficacy. In addition, many human studies are cross-sectional or involve short-term interventions, and substantial inter-individual variability in microbial composition, immune status, and lifestyle factors further complicates the interpretation of findings.

Future research should therefore be grounded in precise phenotyping of constipation subtypes and host characteristics, and promote coordinated design between mechanistic and clinical studies. By combining longitudinal cohort analyses with interventional studies, and integrating microbiome profiling, immune phenotyping, and motility assessments, it will be possible to stratify and validate key mechanistic pathways. Such an approach may ultimately enable more precise identification of target populations and intervention strategies, thereby improving the interpretability and consistency of microbiota-based therapies in constipation management.
